# Surgical outcomes of delayed clipping in ruptured intracranial aneurysms of anterior circulation: Experience from a low-middle-income country

**DOI:** 10.12669/pjms.40.12(PINS).11273

**Published:** 2024-12

**Authors:** Tariq Imran Khokar, Zulqarnain Akram Cheema, Ibreeza Fatima, Maimoona Riaz, Haseeb Mehmood Qadri, Asif Bashir

**Affiliations:** 1 Dr. Tariq Imran Khokar, Associate Professor, Department of Neurosurgery, Unit-I, Punjab Institute of Neurosciences, Lahore, Pakistan; 2 Dr. Zulqarnain Akram Cheema, Post Graduate Resident, Department of Neurosurgery, Unit-I, Punjab Institute of Neurosciences, Lahore, Pakistan; 3 Dr. Ibreeza Fatima, Post Graduate Resident, Department of Neurosurgery, Unit-I, Punjab Institute of Neurosciences, Lahore, Pakistan; 4 Dr. Maimoona Riaz, Post Graduate Resident, Department of Neurosurgery, Unit-I, Punjab Institute of Neurosciences, Lahore, Pakistan; 5 Dr. Haseeb Mehmood Qadri, Post Graduate Resident, Department of Neurosurgery, Unit-I, Punjab Institute of Neurosciences, Lahore, Pakistan; 6 Prof. Dr. Asif Bashir, Department of Neurosurgery, Unit-I, Punjab Institute of Neurosciences, Lahore, Pakistan

**Keywords:** Aneurysm, Subarachnoid haemorrhage, Pakistan, Surgical clip

## Abstract

**Objective:**

To evaluate the surgical outcomes of delayed microsurgical clipping for ruptured intracranial aneurysms (RICAs) of anterior circulation.

**Methods:**

This retrospective, cross-sectional study assessed the surgical outcomes of 50 patients who underwent surgical clipping for “ruptured aneurysms” with subarachnoid haemorrhage after the 21^st^ post-bleed day, from May 01, 2022, till May 01, 2023, at the Department of Neurosurgery, Punjab Institute of Neurosciences, Lahore, Pakistan.

**Results:**

The mean age of patients was 49.66 ± 6.231 years with a female preponderance of 54%. Out of 50 cases, 21 were midline aneurysms (42%), followed by left-sided laterality in 16 cases (32%). Aneurysm of the anterior communicating artery was the most common accounting for 42% of the patients. Forty-nine patients returned home after first postoperative week without deficits, with a success rate of 98%. Seizures, surgical site infection and cerebrospinal fluid leak were noted in 6%, 4% and 2% patients, respectively.

**Conclusion:**

Delayed clipping in RICAs after the 21^st^ post-bleed day is advocated, especially in resource-constrained settings. Meticulous surgical technique and asepsis are responsible for great postoperative outcomes.

## INTRODUCTION

Subarachnoid haemorrhage (SAH) is a form of stroke with an annual reported incidence of 9 per 100,000. The most common cause of SAH is a ruptured aneurysm in 85% of cases.^1^ The acknowledged intracranial complications of aneurysmal SAH include rebleeding, vasospasm, hydrocephalus, seizures and delayed cerebral ischemia, with rebleeding contributing to as much as 70% of cases of mortality.^2^ To prevent this immediate deadly sequel, an aneurysm should be cut off from the remaining cerebral vasculature. Microsurgical aneurysm clipping is possible by open surgery and endovascular intervention.^3^ Endovascular approach is preferred over open microsurgical clipping, but the latter is cheaper and more suited to complex cerebral vasculature, profound vessel tortuosity and aneurysms with underlying hematomas.^3^

Chang et al. compare the hospital costs of open technique versus endovascular intervention in unruptured intracranial aneurysms. Neurosurgical clipping is cheaper than endovascular coiling and reduces hospital costs significantly, however, it is associated with a prolonged hospital stay.^4^ Pakistan is enlisted under the category of low-middle-income countries (LMICs) according to the World Bank and our health system is under financial strain.^5^ Hence, neurosurgical clipping is best suited to our resources for ruptured intracranial aneurysms. It is being offered at a couple of public sector hospitals where the impoverished have a reach.

A large randomized controlled trial from the Netherlands studied 2143 patients for coiling vs. clipping and their outcomes at various days post-SAH, with respect to delayed cerebral ischemia. Authors conclude that surgical clipping is highly advocated at its earliest after aneurysm rupture and SAH while clipping any day after the 10^th^ post-bleed day is associated with worse outcomes.^6^ This study is perhaps the first from Pakistan, an LMIC, documenting the outcomes of clipping for ruptured aneurysms presenting beyond 21^st^ post-bleeding day.

## METHODS

This retrospective, cross-sectional study was conducted at the Department of Neurosurgery, Punjab Institute of Neurosciences (PINS), Lahore, Pakistan in May 2023. We analyzed the records of 50 patients who underwent surgical clipping for “ruptured aneurysm” with subarachnoid haemorrhage from May 01, 2022, till May 01, 2023, irrespective of their age and gender. PINS is a public-sector tertiary care hospital. It caters a large number of patients from all across Pakistan and neighboring countries presenting on variable number of days with subarachnoid hemorrhage. Of all the ruptured intracranial aneurysms (RICA) of anterior circulation clipped at our institution, we aimed to retrospectively analyze the patients presenting beyond 21^st^ post-bleeding day, in terms of their patterns of presentation, intraoperative findings and postoperative complications. Patients with aneurysms of posterior circulation and size greater than 2.5cm, were referred to endovascular neurosurgery for coiling and were therefore excluded from the study.

The information on patient’s demographics, risk factor stratification, pre-operative investigations, intraoperative findings and postoperative complications were collected and analyzed via Google Form (Google Inc., Mountainview, CA) and Microsoft Excel Sheet (Microsoft Corporation, Washington, United States), respectively which was later sent to a statistician for descriptive analysis. Frequency and percentage were calculated for categorical data and mean with standard deviation was computed for continuous data. The study was exempted from ethical approval by the Institutional Review Board of the mentioned hospital, reference # 1754/IRB/PINS/Approval/2024.

## RESULTS

**Fig.1:**
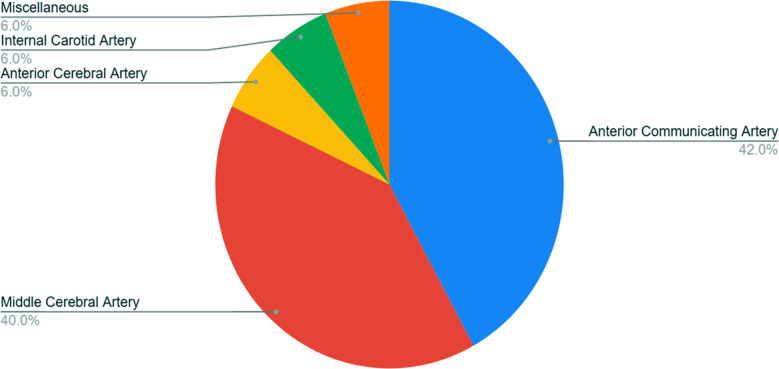
Common sites of occurrence of intracranial aneurysms, where N=50.

Fifty patients fell in the age range of 15 to 76 years, with the mean age of 49.66 ± 6.231 years. Females were 54% of the studied sample, while 46 % were males. The most commonly operated aneurysms were those affecting the anterior communicating artery. Miscellaneous cases included bilateral aneurysms of multiple arteries of anterior circulation (Fig.1).

**Fig.2:**
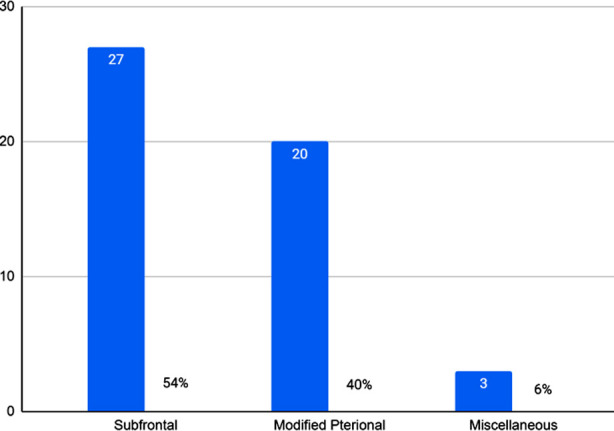
Surgical approaches used for intracranial aneurysm clipping, where N=50.

Out of 50 cases, 21 were midline aneurysms (42%), followed by left-sided laterality in 16 cases (32%) and right-sided aneurysms in 13 cases (26%). Categorically, 50% of MCA aneurysms were left-sided and 50% were right-sided. About 67% of anterior ACA and ICA aneurysms were also left-sided. The gender-wise predilection of laterality of aneurysms is depicted for individual arteries in Table-I. Frontal craniotomy with a subfrontal approach was the most frequently used technique (Fig.2).

**Table-I:** Gender-wise predilection of laterality of intracranial, anterior circulation aneurysms.

Site of Aneurysms	Number of Female Patients	Percentage Occurrence	Number of Male Patients	Percentage Occurrence
Anterior Communicating Artery	12	57.14%	9	42.86%
Middle Cerebral Artery	12	60%	8	40%
Internal Carotid Artery	1	33.33%	2	66.67%
Anterior Cerebral Artery	1	33.33%	2	66.67%
Miscellaneous	1	33.33%	2	66.67%

**Fig.3:**
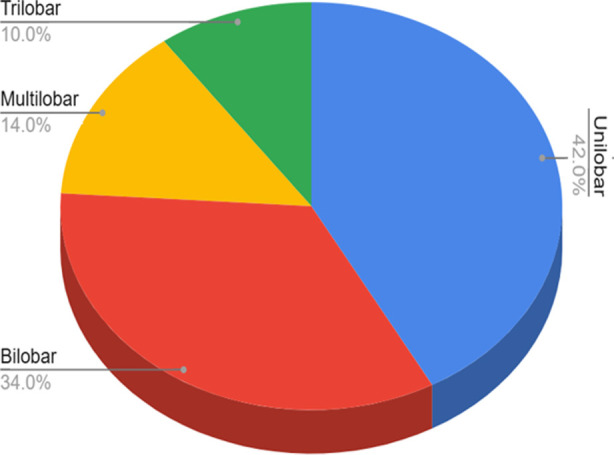
Lobularity of ruptured anterior circulation aneurysms, where N=50.

The majority of patients had hypertension as their existing co-morbidity accounting for 62 % of the patients. Diabetes mellitus and smoking accounted for about 14% and 4% respectively. The most common clinical manifestation at presentation was headache, i.e. in 96% of cases (Table-II). All of the fifty patients presented to our institute beyond 72 hours. Operated cases of aneurysms were graded according to three systems, as shown in Table-III. The most common morphology of aneurysms was their single lobe in 42% of cases (Fig.3).

**Table-II:** Common clinical manifestations at presentation, where N=50.

Clinical Manifestations	Number of Cases, n	Percentage Occurrence
Headache	48	96%
Loss of consciousness	26	52%
Vomiting	21	42%
Seizures	7	14%
Photophobia	2	4%
Neurologic deficit – motor type	2	4%

**Table-III:** Grading of aneurysms according to the three commonly used systems, where N=50.

Aneurysm Grades	Number of cases, n and percentage occurrence, %	I	II	III	IV	V
Hunt and Hess (HH)	n	15	15	20	-	-
	%	30%	30%	40%	-	-
World Federation of Neurosurgical Societies (WFNS)	n	40	10	-	-	-
	%	80%	20%	-	-	-
Modified Fischer	n	50	-	-	-	-
	%	100%	-	-	-	-

Only one patient had an intraoperative re-rupture of aneurysm and survived hydrocephalus after ventriculoperitoneal shunting post-operatively. Forty-nine patients were discharged home without complications after one week, implying a success rate of 98%. Patients were followed in the outpatient department fortnightly for one month and then during third month. Three patients (6%) developed epileptic seizures immediately post-procedure. Two patients (4%) developed surgical site infection and one patient (2%) had cerebrospinal fluid leak which resolved with intravenous antibiotic therapy and reinforcement stitches, respectively.

## DISCUSSION

This study highlights the intervention and outcomes of aneurysmal clipping in a delayed setting. To the best of our literature search using PubMed and Scopus, only three research studies exist from Pakistan, studying the outcomes of surgical management of ruptured aneurysms (Table-IV). Clipping for unruptured anterior circulation aneurysms is not rampant in Pakistan. The focus of clinical burden is microsurgical clipping of ruptured intracranial aneurysms (RICAs) of anterior circulation.^7^-^9^

**Table-IV:** Summary of Published Studies from Pakistan on Microsurgical Clipping of Intracranial Aneurysms.

Study by Year	Study Title	Study Duration	Cohort Size for Clipping	Age (years)	Common Site of Aneurysms
Wadd et al.^7^(2015)	Aneurysmal Subarachnoid Hemorrhage: Outcome of Aneurysm Clipping Versus Coiling in Anterior Circulation Aneurysm	2010 – 2013	70	Mean: 51.00 ±10	Anterior circulation – 100%
Tahir et al.^8^(2009)	Cost-effectiveness of clipping vs coiling of intracranial aneurysms after subarachnoid hemorrhage in a developing country - a prospective study	2004 – 2007	31	Average: 45	Anterior circulation – 83.64%
Ahmad et al.^7^(2008)	Clipping of intracranial aneurysms - 3 years study	2003 – 2005	75	Range: 40 – 60	Anterior circulation – 78.3%

The mean age of 50 patients included in our study was 49.66 ± 16.23 years. A study conducted by Sobti et al. showed a mean age of 52.43 ± 8.6 years while describing the clinical-radiological profile of patients with RICAs. This is explained by the fact that the incidence of RICAs is directly proportional to advancing age, although no clear reasons for this association exist.^10^,^11^

We found a higher prevalence of aneurysms in females compared to males in our research. The findings of a giant Japanese study with 2037 patients are consistent with our results, suggesting female gender is an important risk factor in the occurrence and pathogenesis of intracranial aneurysms across all age groups, as compared to men.^12^ A study in 2016 also demonstrates a female preponderance of 54.1% of cases of intracranial aneurysms.^3^ Two possible explanations exist for this observation, in general: females have a smaller diameter of brain blood vessels, leading to faster blood flow and higher wall shear stress at key branching points in the cerebral arteries, leading to mural weakness. A decline in oestrogen levels in postmenopausal women makes vessels more susceptible to damage as it maintains collagen integrity normally.^12^,^13^

There was a 42% and 40% occurrence of aneurysms of the anterior communicating artery (ACOMA), followed by the middle cerebral artery (MCA), respectively. This implies the almost similar prevalence of ACOMA and MCA aneurysms at our center. Contrastingly, a study on the comparison of surgical techniques for intracranial clipping by Sik Park and his colleagues highlights MCA aneurysms as the most common aneurysms in 56.37% of patients.^14^ Females had more aneurysms of ACOMA and MCA than males at our center, which is surprisingly different from a recent topical review by Feuntes et al. It demonstrates a difference in the distribution of intracranial aneurysms between genders, with men having a higher incidence of anterior cerebral artery (ACA) and ACOMA aneurysms and women having a higher incidence of internal carotid artery (ICA) aneurysms. These findings suggest that hormonal differences and gender-specific shear stress may play a role in the formation of intracranial aneurysms.^15^

In our study, the majority of the patients had hypertension, followed by diabetes mellitus and smoking as their existing comorbidities. Similarly, an Indian cohort study conducted by Singh et al. had 68% of patients with hypertension and 24% of patients had diabetes mellitus.^16^ The development of aneurysms may be influenced by several genetic and environmental factors that directly impact the strength of arterial walls. Jin and colleagues have documented an association between hypertension and the chances of rupture of intracranial aneurysms, but the exact reasons are still to be sought.^17^

The most common clinical manifestations at presentation were headache in almost all patients, followed by loss of consciousness, vomiting and seizures in our cohort. Singh et al. report similar presenting complaints of headache, altered sensorium and seizures. The reason for this difference is that brain aneurysms can have different clinical manifestations depending upon the location, size, and severity of the aneurysm, as well as the age and overall health of the patient.^16^

The Hunt and Hess scale (HHS) is used to predict prognosis and outcome in patients with subarachnoid haemorrhage. A higher grade predicts a poor outcome. In our study, the majority of patients presented with HHS Grade II and III, similar to another study with 40% of patients in Grade II.^16^ A retrospective study in Nigeria showed that 58.6% had a pre-operative HHS Grade III and 48.3% of patients had a WFNS Grade III which implicated a higher number of patients present with higher grades in resource-limited settings.^18^

Various studies have shown that the World Federation of Neurosurgical Societies (WFNS) grade has been shown to be a crucial factor in predicting outcomes, with patients presenting with better grades having better chances of recovery.^10^,^19^ The present study further supports these findings by showing that patients with good WFNS grades at presentation had significantly better outcomes than those with worse grades. In our study, patients with WFNS Grade I and II comprised 80% and 20% of the total sample, respectively, while Sobti et al. had 50.9% of their patients with WFNS Grade 1.^10^ The direction of aneurysms of the anterior communicating artery is a crucial factor in predicting the difficulty in the microsurgical clipping of intracranial aneurysms. Aneurysms of the anterior communicating artery can be anterosuperior, posterosuperior, anteroinferior or posteroinferior.^20^ There were an equal number of cases, 34% each with anteroinferior and posterosuperior direction of aneurysms in our study. This contrasts with the tertiary care experience of Indian authors where 32.3% of aneurysms had anterosuperior direction and 6.5% had posterior direction.^10^

Forty-nine out of 50 patients were discharged home on their seventh postoperative day, free of complications at our centre implying a high success rate. One patient needs a special mention, an eleven-year-old boy had a ruptured ICA aneurysm, which re-ruptured intra-operatively. The RICA was however clipped successfully. He developed hydrocephalus on the third postoperative day and had a ventriculoperitoneal shunt later on.

It is important to highlight here that most of our patients were from rural and outstation areas of Pakistan and they tend to arrive late (particularly after days). Glittins et al., in his cohort of 111 patients, implied that immediate management was associated with lower disability at discharge than delayed management, but this difference did not persist after three months. Overall, immediate management and delayed management after poor-grade subarachnoid bleed are associated with similar morbidity and mortality at 12 months.^21^

Although the long-term follow-up of our patients is still pending, only a few patients developed immediate postoperative complications (CSF leak, seizures, hydrocephalus) which could imply that despite the current literature is more towards early clipping of aneurysm, in an LMIC, delaying clipping is still a possibility, especially in poor-grade subarachnoid bleeds. Therefore, it may be a reasonable approach to delay intervention in poor-grade patients especially if there is a need for time to plan the procedure or stabilize the patient.^18^

**Fig.4:**
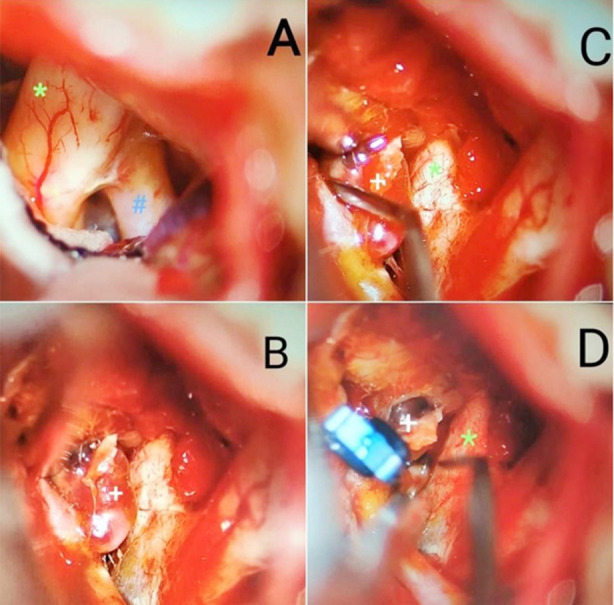
Intraoperative view of clipping of aneurysm of the anterior communicating artery. **A.** Optic nerve shown in green asterisk and internal carotid artery in blue hash; **B.** White plus denoting aneurysmal sac; **C.** Dissection of aneurysm being done; **D.** Permanent clip applied to the neck of aneurysm, with adjacent optic nerve in situ.

The satisfactory success rates of delayed microsurgical clipping of ruptured aneurysms at our hospital clearly implies that the optimal timing to perform clipping is still controversial and the optimum time to clip can be extended depending on the condition of the patient and the severity of the disease (Fig.4).

### Limitations:

The small sample size combined with the retrospective nature of the study and the short follow-up duration of our patients are the major factors of limitation and hence results cannot be generalized. Multicentric studies are a requirement on this topic.

## CONCLUSION

Intracranial aneurysms have variable patterns of presentation worldwide, though female gender and increasing age are closely associated with chances of rupture and symptomatic presentation. Microsurgical clipping can be preferred in resource-limited settings over endovascular coiling for RICAs. Although the results of clipping in late-presenting patients are still promising and can be applied in selected patients who present late to tertiary health facilities, the long-term sequelae, and complications of such interventions still need thorough follow-up. The choice of surgical approach depends on the surgeon’s preference, patient characteristics, presentation, and the location and size of the aneurysm. Meticulous surgical technique and asepsis are responsible for great postoperative outcomes.

### Authors Contribution:

**TIK** conceived the study, prepared the manuscript draft, critically reviewed and is responsible and accountable for the accuracy or integrity of the work.

**ZAC, IF and MR** contributed to literature review, data collection and manuscript writing.

**HMQ** designed the study, did literature search, wrote the manuscript draft and critically reviewed the article.

**AB** supervised the project and critically reviewed the article.

All authors have approved the final version of the manuscript.
